# Belladonna in Tetanus

**Published:** 1844-07-13

**Authors:** 


### BELLADONNA IN TETANUS.

Dr. Hutchinson narrates a case of tetanus, apparently traumatic, treated by Dr. Williams, by his advice, with a large dose of extract of belladonna, (five grains,) with evident benefit. The man ultimately recovered. Dr. Williams says he has not always found belladonna a successful remedy, either in pure or rheumatic neuralgia, and that it must be used with great caution and watchfulness. He has found the influence of belladonna very variable in different constitutions, some being extremely susceptible of it. It ought never to be continued when the pupils become dilated, and a very long experience has taught him, he says, never to prescribe it internally or externally, if there be any amaurotic disease. He has seen it act as a stimulus to the brain, producing pain in the eyeballs, intolerance of light, and conjunctival inflammation; these symptoms soon followed by dilated pupils and loss of sight, paralysis of the iris, and the blindness being permanent. A few months since a friend of his painted over the forehead of a delicate lady patient a fluid extract of belladonna, for a severe neuralgic pain in the head. In about three hours the pain was relieved, and on his return the application was renewed, the intervals of pain were longer, and the pain less severe; after the fourth or fifth application it ceased, the pupils became dilated, and vision imperfect, vomiting and purging were induced; there were also perceived great weakness on the left side, numbness of the left side of the face and arm, and a pricking sensation in the same parts. The left pupil remained more than two months dilated, and the sight impaired; the numbness and pricking sensations have subsided, but the left arm has scarcely yet recovered its strength.—*Ibid.*

